# 

**Published:** 1898-11

**Authors:** 


### Books Received.

White's Materia Medica and Therapeutics. Wilcox. P. Blakiston's Son & Co.

Handbook of Practice of Medicine. Taylor. P. Blakiston's Son & Co.

Osler's Practice of Medicine, Third Edition. D. Appleton & Co.

Practical Urinalysis and Urinary Diagnosis. By Charles W. Purdy, M.D., LL.D., Fourth Edition. The F. A. Davis Com, pany, Philadelphia.

Primer of Psychology and Mental Disease. C. B. Burr, M.D., second revised edition. The F. A. Davis Company. Philadelphia.

Physicians' Visiting List for 1899. P. Blakiston's Son & Co., Philadelphia.

Morris' Anatomy, Second Edition. P. Blakiston's Son & Co.

